# The Silent Invaders: Microplastic Accumulation, Impacts, and Monitoring Approaches

**DOI:** 10.3390/toxics13030192

**Published:** 2025-03-07

**Authors:** Grigorios L. Kyriakopoulos, Antonis A. Zorpas, Vassilis J. Inglezakis, Rocío Rodríguez-Barroso

**Affiliations:** 1 Photometry Laboratory, Electric Power Division, School of Electrical and Computer Engineering, National Technical University of Athens, 9 Heroon Polytechniou Street, 15780 Athens, Greece; 2 Laboratory of Chemical Engineering and Engineering Sustainability, Faculty of Pure and Applied Sciences, Open University of Cyprus, Giannou Kranidioti 89, Latsia, 2231 Nicosia, Cyprus; antonis.zorpas@ouc.ac.cy; 3 Department of Chemical & Process Engineering, University of Strathclyde, Glasgow G1 1XJ, UK; vasileios.inglezakis@strath.ac.uk; 4 Department of Environmental Technologies, Faculty of Marine and Environmental Sciences, University of Cádiz, 11510 Cádiz, Spain; rocio.rodriguez@uca.es

## 1. Introduction

Environmental pollution caused by microplastics (MPs) has evolved into a global concern; however, the knowledge about MP accumulation in the environment, potential impacts, and monitoring approaches is limited. MPs, defined as plastic particles smaller than 5 mm, have emerged as pervasive pollutants infiltrating ecosystems, food chains, and even human bodies according to several reports. MPs are now recognized as a major environmental challenge due to their microscopic size, with far-reaching consequences. This editorial summarizes the current knowledge on MP accumulation, impacts, and monitoring approaches.

MPs are plastic particles less than 5 mm in diameter coming from commercial products such as cosmetics and microfibers in textiles and the degradation of larger plastic waste in the environment. They have been recognized as a major environmental and human health hazard. This is because MPs take a long time to decompose, they are widespread, and they find their way into the food chain through consumption by marine animals. Also, they will continue to be released into the environment since plastic usage is widespread. Plastics are amazing products with infinite applications, such as food packaging, the aviation and automotive industry, pharmaceuticals, medicine, telecommunication, apparel industry, etc. Nafea et al. [1] pointed out that global plastic production has doubled since 2000, reaching 460 million tonnes in 2019, and at the same time, plastic waste generation reached 353 million tonnes. Regrettably, only 9% of all plastics produced are recycled, with 68% ending up in landfills or being incinerated and 22% being mismanaged and entering the natural environment [2]. Chlorinated plastics and MPs can release harmful chemicals into the soil, which can then seep into groundwater or other water sources. Landfill areas are constantly piled high with many different types of plastics, and there are many microorganisms which speed up their biodegradation, releasing methane.

Due to the highly complex nature of plastic pollution, encompassing a wide range of polymers, various products and packaging, diverse societal uses, and numerous pathways into different environmental compartments [3], monitoring approaches are equally challenging. Each sector of plastic usage in society must implement customized solutions to address the specific polymers, products, and packaging it utilizes, as well as their potential release into the environmental media.

At the same time, plastic pollution is a defining characteristic of modern society, and its ubiquity has been noted by scientists from every continent. Researchers have found plastic particles in the deep sea [4], rainwater [5], on top of mount Everest [6], and even in human placenta [7], blood [8], drinking water [9], plankton [10], coral reef [11], and sea mammals and birds [12]. Despite the increased attention given to plastic pollution in the last decade, evidence suggests that the problem is worsening [13].

To combat plastic pollution, countries have begun regulating the production, consumption, and distribution of plastics. The European Commission (EC) is actively implementing initiatives from the European Green Deal and the newly introduced Circular Economy Action Plan. Several directives, including the Packaging and Packaging Waste Directive and the Waste Framework Directive, and the ban on single-use plastics, are being enforced to tackle this problem. The issue of plastic pollution, and therefore MPs, is not strictly economic or environmental pollution, but it can be said to reflect environmental justice [13]. Innovative strategies are essential, requiring a comprehensive understanding of plastic pollution, especially its root causes and the role of global sustainability agendas in addressing the issue. A strategic approach to mitigate pollution (including plastic pollution) was proposed by Zorpas [14]. In general, plastic pollution can be reduced by adopting several R strategies [15]. These methods reflect circular economy principles, SDGs, and the new circular economy framework standard ISO 59000 series [15].

There are several technologies for the plastic clean-up of seas, lakes, rivers, oceans, and shores, but they are insufficient in addressing the vast extent of microplastic pollution [1]. Moreover, to the best of the authors’ knowledge, there are no technologies for the removal of MPs from agricultural lands or forests. Hence, effective plastic waste management, policies, and legislation play a crucial role in reducing environmental plastic and microplastic pollution. Regulations can encourage research, innovation, and adoption of sustainable practices leading to a more circular resource-efficient approach to plastic and microplastic management [16]. For example, the European policy “Strategy for Plastics in a Circular Economy” aims to establish a new circular economy for plastics across the continent, promoting the reuse, repair, and recycling of plastics and plastic products while striving to reduce the introduction of microplastic products. In general, proper waste management practice can mitigate the negative impacts of plastic pollution on the environment.

## 2. Research Topic Overview

The Research Topic titled “Microplastics Pollution” contains 26 articles that have been published by the following five participating MDPI journals: *International Journal of Environmental Research and Public Health*, *Microplastics*, *Sustainability*, *Toxics*, and *Water*. These articles are presented below:

“Enhancing Pb Adsorption on Crushed Microplastics: Insights into the Environmental Remediation”, by Sen Li, Lu Cao, Qiyuan Liu, Shuting Sui, Jiayin Bian, Xizeng Zhao, Yun Gao. This study investigates the pollution characteristics and environmental risks of crushed microplastics (MPs) generated during plastic recycling, emphasizing their adsorption capacity for heavy metals, particularly lead (Pb), by employing a variety of advanced analytical techniques, such as that of SEM-EDS, revealing that crushed MPs exhibit significantly higher adsorption capacity than primary MPs. Nevertheless, the presence of adsorbed Pb slightly reduced recovery performance, emphasizing the need to optimize recovery conditions for maximum efficiency. Therefore, the study demonstrated the dual threat posed by crushed MPs: their capacity to both adsorb and concentrate harmful substances, and thus, the increase in ecological toxicity and challenge to their recovery.

“Evaluation of Microplastic Toxicity in Drinking Water Using Different Test Systems”, by Natalya S. Salikova, Anna V. Lovinskaya, Saule Zh. Kolumbayeva, Ainash U. Bektemissova, Saltanat E. Urazbayeva, María-Elena Rodrigo-Clavero, Javier Rodrigo-Ilarri. This study investigated the toxicological and genotoxic effects of various microplastic types (polystyrene (PS), polyethylene terephthalate (PET), polypropylene (PP), and polyethylene (PE)) on plant and animal models. The findings of this study underscored the need to mitigate risks associated with microplastic pollution, particularly in drinking water sources. Future research works could closely approach the long-term health impacts of microplastic exposure, including carcinogenic potential coupling with the exploration of the synergistic effects of other pollutants.

“Microplastic Transport and Accumulation in Rural Waterbodies: Insights from a Small Catchment in East China”, by Tom Lotz, Wenjun Chen, Shoubao Su. This study investigates the distribution and sources of MP in drainage ditches influenced by pond connectivity, land use, and soil properties. Interestingly, site-specific MP sources have been determined, with fluororubber (FR) linked to road runoff and polyethylene terephthalate (PET) being suitable for agricultural practices. Correlations between MP shape and soil properties proved that more compact and filled shapes were more commonly associated with coarser soils. In this context, PE particle size was negatively correlated with organic matter. The most general applicability issues of this study refer to the need for targeted strategies in order to reduce MP pollution in rural landscapes, including reduction in plastic use, ditch maintenance, and improved road runoff management.

“Sustainable Wastewater Treatment Strategies in Effective Abatement of Emerging Pollutants”, by Hafiz Waqas Ahmad, Hafiza Aiman Bibi, Murugesan Chandrasekaran, Sajjad Ahmad, Grigorios L. Kyriakopoulos. This study underscores how the fundamental existence of any living organism requires the availability of pure and safe water. In this context, this study is a review analysis on MPs, considering the ever-increasing population that has led to extensive industrialization and urbanization, which have subsequently escalated micropollutants and water contamination, as well as the MPs’ environmental impact on various life forms. Environmentally friendly, cost-effective, and sustainable strategies are envisaged for efficient environmental protection. The review study also proposed a novel strategy that combines nanomaterials to improve micropollutant degradation with bioremediation techniques, particularly the creative application of phytoremediation technologies like floating wetlands. The critical domains of water pollution abatement included floating wetland treatments, facilitating micropollutant elimination, landscape management, ecosystem conservation, and aesthetic enhancement in diverse environments. The novelty of this research focus includes the incorporation of nanomaterials in the bioremediation of toxic micropollutants, augmenting novel and innovative strategies.

“Retention, Degradation, and Runoff of Plastic-Coated Fertilizer Capsules in Paddy Fields in Fukushima and Miyagi Prefectures, Japan: Consistency of Capsule Degradation Behavior and Variations in Carbon Weight and Stable Carbon Isotope Abundance”, by Shigeki Harada, Itsuki Yajima, Keitaro Fukushima, Youji Nitta. In this study, paddy field runoff containing plastic capsules that are used to coat fertilizers has been receiving increased attention. However, the behavior of these capsules, especially their degradation behavior, has not been extensively investigated, which was the research focus of this study. The behaviors of the capsules in both types of runoff were monitored in 2022 and 2023 at four paddy fields in Fukushima and Miyagi prefectures in northern Japan. Weight loss of the plastic capsules could be attributed to a combination of capsule degradation and the release of urea inside the capsules, which was explained by carbon isotopic analyses. The following types of degraded capsules were identified: shrunken, broken, and spherical, making it statistically significant to signify the differences among the weights of each type.

“Sorption-Based Removal Techniques for Microplastic Contamination of Tap Water”, by Natalya S. Salikova, Almagul R. Kerimkulova, Javier Rodrigo-Ilarri, Kulyash K. Alimova, María-Elena Rodrigo-Clavero, Gulzhanat A. Kapbassova. This study investigates the presence of MPs in tap drinking water and evaluates the efficacy of various sorbents for their removal in the context of Kazakhstan’s water treatment system. Water samples were taken in the cities of Kokshetau and Krasny Yar (Akmola Region). The research objective of this study was the sorption efficiency of different sorbents, indicating especially high retention rates (ranging from 82.7 to 97.8%) for microplastic particles of different shapes and synthesis. Indeed, sorbents synthesized by carbon sorption material (CSM) demonstrated the highest efficiency in both microplastic retention and improvement in water quality parameters, making it a promising technology especially suitable for water treatment facilities and household filters.

“Spatial and Temporal Distribution Characteristics and Potential Sources of Microplastic Pollution in China’s Freshwater Environments” by Hualong He, Sulin Cai, Siyuan Chen, Qiang Li, Pengwei Wan, Rumeng Ye, Xiaoyi Zeng, Bei Yao, Yanli Ji, Tingting Cao, Yunchao Luo, Han Jiang, Run Liu, Qi Chen, You Fang, Lu Pang, Yunru Chen, Weihua He, Yueting Pan, Gaozhong Pu. This study investigated the characteristics of microplastic pollution in the freshwater environments of 21 major cities across China. Through indoor and outdoor experimental analysis, their spatial and temporal distribution characteristics were identified. Authors observed that the abundance of MPs in China’s freshwater environments was generally increased from west to east and from south to north, while other factors of positive correlation were that of (a) areas of intense human activity, including agricultural, transport, and urban land, (b) seasonal changes peaking in summer, followed by spring and autumn, and (c) rainfall variations.

“Microplastics in Groundwater: Pathways, Occurrence, and Monitoring Challenges”, by Elvira Colmenarejo Calero, Manca Kovač Viršek, Nina Mali. In this study, MPs are considered a fast-emerging pollutant, whose presence in the water cycle and interaction with ecological processes pose a significant environmental threat to surface and groundwater, which represent the primary sources of drinking water. Monitoring MPs in groundwater (GW) is determined primarily by the contamination pathways of MPs from surface water, seawater, and soil into the GW. Such an analysis identified the challenges associated with their monitoring in GW, among which (challenges) are that of applying standardized techniques for MP sampling and detection, by better understanding the specific hydrogeological and hydrogeographic conditions in the process of sample collection using sampling devices with comparable specifications and comparable laboratory techniques for MPs’ identification.

“Monitoring of Microplastics in Water and Sediment Samples of Lakes and Rivers of the Akmola Region (Kazakhstan)”, by Natalya S. Salikova, Javier Rodrigo-Ilarri, Lyudmila A. Makeyeva, María-Elena Rodrigo-Clavero, Zhulduz O. Tleuova, Anar D. Makhmutova. This study provides a detailed description of the findings and methodology related to the monitoring of MPs in three lakes and one river of the Akmola Region in Kazakhstan. The research variables were the concentration of microplastic particles and the connection between microplastic content and turbidity, particularly notable during the spring season. Sediment analysis revealed a decrease in microplastic concentrations from the coastal zones. In addition, the spatial and temporal distribution of MPs necessitated the ongoing monitoring and management strategies to address emerging environmental concerns.

“Comparison of Methodologies for Microplastic Isolation through Multicriteria Analysis (AHP)”, by Valentina Phinikettou, Iliana Papamichael, Irene Voukkali, Antonis A. Zorpas. This study developed an inexpensive, rapid method with user-friendly and environmentally sustainable outcomes, considering the limited knowledge that exists about MPs in soils due to the absence of standardized extraction methods. In better understanding the mitigation of MPs in soils, a comprehensive multicriteria analysis proved that saturated sodium chloride solution emerged as the optimal scenario for MP extraction in terms of demonstrated economic feasibility, safety, and reliability, followed closely by the canola oil scenario.

“Interaction between Microplastics and Pathogens in Subsurface System: What We Know So Far”, by Hongyu Zhao, Xiaotao Hong, Juanfen Chai, Bo Wan, Kaichao Zhao, Cuihong Han, Wenjing Zhang, Huan Huan. This study has focused on MPs that are abundant in soil and the subsurface environment. Authors stated that MPs can co-transport with pathogens or act as vectors for pathogens, potentially causing severe ecological harm. The interaction of MPs with pathogens is environmentally important, especially when focusing on interaction mechanisms and environmental factors and their co-transport. Such environmental factors affecting their interaction include particle size, specific surface area, shape and functional groups of MPs, the zeta potential and auxiliary metabolic genes of pathogens, and hydrophobicity.

“Application and Efficacy of Management Interventions for the Control of Microplastics in Freshwater Bodies: A Systematic Review”, by Suveshnee Munien, Puspa L. Adhikari, Kimberly Reycraft, Traci J. Mays, Trishan Naidoo, MacKenzie Pruitt, Jacqueline Arena, Sershen. This study is a review analysis that systematically represents one of the first attempts to trace the evolution of research and compare the efficacy of the full suite of management interventions developed to control (prevent or remove) MPs in freshwater bodies, both man-made and natural. Among the key findings of this review paper are that physical types (particularly membrane filtration) were most common. It is notable that wastewater/sludge, stormwater, and in situ water/sediment categories exhibited removal efficacies of more than 90% under laboratory conditions. It is also questionable whether scalability and suitability could be achieved across different settings. Moreover, downstream interventions lack sustainability without effective upstream interventions, and when in situ methods are technically achievable, they may not be feasible in resource-limited settings.

“Release of Microplastics from Urban Wastewater Treatment Plants to Aquatic Ecosystems in Acapulco, Mexico”, by Enrique J. Flores-Munguía, José Luis Rosas-Acevedo, Aurelio Ramírez-Hernández, Alejandro Aparicio-Saguilan, Rosa M. Brito-Carmona, Juan Violante-González. This study denotes that contamination of aquatic ecosystems by MPs is mainly due to the release of high levels of microplastic particles from treated effluents by wastewater treatment plants (WWTPs). The authors also point out the lack of policies and regulations establishing criteria for the control and elimination of hazard pollutants from aquatic ecosystems, such as that of Acapulco, Mexico, which is the research area of their study. The MPs’ average daily emissions to the receiving bodies of the three selected WWTPs ranged from 9.5 × 10^6^ to 4.70 × 10^8^ particles, while the annual emissions ranged from 3.05 × 10^9^ to 1.72 × 10^11^ particles. The generalized prospects are relevant to the urgency of implementing regulatory policies to avoid the continuous emission of MPs in the studied area and its WWTPs.

“Microplastic in the Snow on Sledding Hills in Green Areas of Krakow”, by Jarosław Lasota, Wojciech Piaszczyk, Sylwester Tabor, Ewa Błońska. This study aims to determine the amount of microplastic in the snow on sledding hills in green areas of Krakow, considering the intensive use of these sledding hills in winter. After the snow melts, microplastics are transferred to the soil surface, which can lead to changes in the properties of the soil. Subsequently, due to their strong hydrophobicity, they will play an important role in the transport of toxic compounds, e.g., polycyclic aromatic hydrocarbons (PAHs). The research objective is to limit the use of plastic sleds and replace them with wooden sleds, which will not be a source of pollution for urban green spaces used by residents regardless of the season.

“Influence of Microplastics on Morphological Manifestations of Experimental Acute Colitis”, by Natalia Zolotova, Dzhuliia Dzhalilova, Ivan Tsvetkov, Olga Makarova. This study demonstrates that microplastic pollution poses a threat to human health, and it is highly possible that the increase in the incidence of inflammatory bowel disease is associated with exposure to MPs. For this reason, authors investigated the effect of the consumption of polystyrene microparticles with a diameter of 5 μm, focusing on the acute colitis body episodes. Exposure of healthy mice to MPs resulted in an increase in endocrine cell numbers, an increase in the content of highly sulfated mucins in goblet cells, an increase in the number of cells in the lamina propria, and a decrease in the volume fraction of macrophages. The extent to which microplastic consumption caused more severe acute colitis can be characterized by a greater prevalence of ulcers and inflammation and a decrease in the content of neutral mucins in goblet cells.

“Environmental Assessment of Microplastic Pollution Induced by Solid Waste Landfills in the Akmola Region (North Kazakhstan)”, by Natalya S. Salikova, Javier Rodrigo-Ilarri, María-Elena Rodrigo-Clavero, Saltanat E. Urazbayeva, Aniza Zh. Askarova, Kuandyk M. Magzhanov. This study presents the outcomes derived from an environmental assessment of microplastic pollution resulting from solid waste landfills in the Akmola Region, situated in North Kazakhstan. This research was conducted on MPs within this specific region. The utilized methodologies were focused on the “soft” removal of organic substances through the use of oxidants which do not damage plastics and were tested using a water-bath therapeutic treatment. Furthermore, an analysis of soil samples taken from the landfills unveiled the ultimate retention of microplastic particles, attributed to leachate and rainwater runoff.

“Spatial–Temporal Distribution and Ecological Risk Assessment of Microplastics in the Shiwuli River”, by Lei Hong, Xiangwu Meng, Teng Bao, Bin Liu, Qun Wang, Jie Jin, Ke Wu. This study aims to investigate the distribution of MPs within the Shiwuli River in Hefei, a Chinese inland city. Water and sediment samples were collected during both flood season (May to September) and non-flood season (October to April) at 10 representative sites to assess the potential risk posed by MP pollution in the Shiwuli River to the quality of drinking water sources in Chaohu Lake in Hefei. The analytical part of this study contained an analysis of the main variables that determine the level of MP existence. Such variables include tributaries that are also close to residential and industrial development, agricultural areas, wetland ecological regions, and flood season or non-flood season. The research findings provide valuable insights into management, pollution control, and integrated management strategies pertaining to MPs in urban inland rivers (with a research focus on Hefei).

“A Review of the Current State of Microplastic Pollution in South Asian Countries”, by Lee Tin Sin, Vineshaa Balakrishnan, Soo-Tueen Bee, Soo-Ling Bee. This study is a review analysis that discusses the development of microplastic pollution based on a selection of South Asian countries consisting of Bangladesh, Iran, Philippines, Thailand, India, Indonesia, and Vietnam. The presence of MPs in food items, mainly tea bags, sugar, shrimp paste, and salt packets, has been reported. Microplastic contamination includes the ingestion of MPs by aquatic creatures in water environments. The impacts on terrestrial environments relate to MPs sinking into the soil, leading to the alteration of the physicochemical parameters of soil. Moreover, the impact on the atmospheric environment included the settling of MPs on the external bodies of animals and humans.

“Assessing the Occurrence and Distribution of Microplastics in Surface Freshwater and Wastewaters of Latvia and Lithuania”, by Reza Pashaei, Viktorija Sabaliauskaitė, Sergej Suzdalev, Arūnas Balčiūnas, Ieva Putna-Nimane, Robert M. Rees, Reda Dzingelevičienė. This study focuses on microplastic concentrations in surface water and wastewater collected from the cities of Daugavpils and Liepaja in Latvia, as well as Klaipeda and Siauliai in Lithuania, which were measured in July and December 2021. In this study, a variety of advanced analytical techniques contained the use of optical microscopy, and polymer composition can be characterized using micro-Raman spectroscopy. Surface water and wastewater samples showed that municipal and hospital wastewater from catchment areas were the main reasons for the contamination of MPs in the surface water and wastewater of Latvia and Lithuania. Reduction in pollution loads can be realistic by implementing measures such as raising awareness, installing more high-tech wastewater treatment plants, and reducing plastic use.

“Social Cognitive Theory and Reciprocal Relationship: A Guide to Single-Use Plastic Education for Policymakers, Business Leaders and Consumers”, by Sarah Fischbach, Brielle Yauney. This study concentrates on the social cognitive theory framework for sustainable consumption. The authors employed a US-based survey in which the reciprocal relationship among three factors—personal (green consumer values), environmental (bans and rebate/reward programs), and behavioral (consumer decision-making related to single-use plastic waste)—was examined. The key findings of this study are that those states with bans or rebate/reward programs tend to have higher green consumer values, and consumers in those states report less use of single-use plastic waste. Education level is a determining factor that impacts green consumer values and plastic waste usage. A resource guide was developed for decision makers to implement programs in five areas, including Business Resources, Public Policy Resources, Non-Profit Resources, Education Resources, and Personal Resources.

“High-Efficiency Microplastic Sampling Device Improved Using CFD Analysis”, by Seonghyeon Ju, Jongchan Yi, Junho Lee, Jiyoon Kim, Chaehwi Lim, Jihoon Lee, Kyungtae Kim, Yeojoon Yoon. In response to the fact that MPs are considered harmful to the human body, studies on their samplings, pre-treatments, and environmental media analyses, such as water, are continuously being conducted. However, there is an imperative need to develop a standard sampling and pre-treatment method, particularly because MPs of a few micrometers in size are easily affected by external contamination. For the evaluation of the developed device, microplastic reference materials were produced and used, and computational fluid dynamics (CFD) analysis was performed. This device has been applied to the relatively large previously studied microplastics (100 µm), but it is also suited to MPs of approximately 20 µm that are vulnerable to contamination. A recovery rate of 94.2% was obtained using this device, and the particles were separated by filtration through a three-stage cassette. Accuracy and reproducibility of results for microplastic contamination in the environment are needed. This method is able to consistently obtain and manage MP data, which are often difficult to compare using various existing methods.

“Occurrence Characterization and Contamination Risk Evaluation of Microplastics in Hefei’s Urban Wastewater Treatment Plant”, by Xiangwu Meng, Teng Bao, Lei Hong, Ke Wu. This study denotes that MPs are one of the primary nodes in their flow through the environment, making it critical to examine and assess sewage treatment, occurrence, and removal of MPs in a waste treatment plant (WWTP). The examined variables contain the shape, size, and composition of MPs at various stages of the WWTP process in the south of the city of Hefei, China, considering both the dry and the rainy weather conditions, as well as the removal effectiveness of MPs in a three-stage process. The methodology and findings of this study are case specific to the examined area. In particular, pollution indices of MPs in row water and tail water were 2.40 and 2.46, respectively, which were heavily contaminated, and 1.0 and 1.2, which were moderately polluted. MPs in dewatered sludge had severely polluted indexes of 3.5 and 3.4, respectively. MP efflux or build-up in sludge during and after the WWTP process presents an ecological contamination concern.

“Experimental Investigation of Water-Retaining and Unsaturated Infiltration Characteristics of Loess Soils Imbued with Microplastics”, by Jiahui Gu, Liang Chen, Yu Wan, Yaozong Teng, Shufa Yan, Liang Hu. This study explores the effect of MPs on agricultural soil permeability by simulating the rainfall irrigation process. For this reason, a one-dimensional vertical soil column rainfall infiltration test device was used to study the unsaturated infiltration characteristics of loss soil imbued with MPs under rainfall conditions. MPs represent negative effects on rainfall infiltration and soil water retention, so it is recommended to dispose of them.

“Assessment of Microplastics in Green Mussel (Perna viridis) and Surrounding Environments around Sri Racha Bay, Thailand”, by Jitraporn Phaksopa, Roochira Sukhsangchan, Rangsiwut Keawsang, Kittipod Tanapivattanakul, Bojara Asvakittimakul, Thon Thamrongnawasawat, Suchai Worachananant. This study analyses the characteristics of MPs in the seawater, sediments, and green mussels (Perna viridis) around Sri Racha Bay, Thailand. In the field of food management and practices this study signified that the excessive riverine freshwater discharge is transported terrestrial plastic debris into the estuarine system, thus, higher microplastic contamination in surface seawater and sediment was evidenced. The presence of colorants in organisms revealed an anthropogenic origin through the use of a wide array of applications.

“Adsorption Behavior of Nonylphenol on Polystyrene Microplastics and Their Cytotoxicity in Human CaCO_2_ Cells”, by Fangfang Ding, Qianqian Zhao, Luchen Wang, Juan Ma, Lingmin Song, Danfei Huang. This study points out that -as two environmental pollutants of great concern, polystyrene microplastics (PS-MPs) and nonylphenol (NP) often coexist in the environment and cause combined pollution- authors carried out batch adsorption experiments by varying parameters such as pH, the particle sizes of the PS-MPs, the initial concentration of NP, and metal ion content. The NP adsorption process of the PS-MPs was optimally matching to pseudo-second-order kinetic model and Langmuir isotherm model, while the intraparticle diffusion and Bangham models were also involved in determining the NP adsorption process.

“Investigating the Epigenetic Effects of Polystyrene Nanoplastic Exposure in Bluegill (*Lepomis macrochirus*) Epithelial Cells Using Methylation Sensitive-AFLPs”, by Sheridan M Wilkinson, Justine M Whitaker, Alexis M Janosik. This study has focused on MPs, defining them as remnants of macroplastics that have broken down to fragments smaller than 5 mm, and nanoplastics, broken down even further to sizes < 1 μm, are pervasive in aquatic ecosystems. The accumulation of plastic in the organ gut can result in various repercussions, including cellular contamination and genomic modifications such as DNA methylation. The study delves into such a largely uncharted territory, investigating the accumulation of methylation due to nanoplastic exposure within the genome of cultured bluegill BF-2 cells (*Lepomis macrochirus*) using methylation-sensitive AFLPs. It is also noteworthy that higher 21 dosages and exposure times to nanoplastics do not result in increased methylation levels in congruence with the dosage and exposure time, but rather only the presence of nanoplastics is enough to cause DNA methylation changes.

Based on the aforementioned framework of the 26 articles, and considering the perspective of opening up a scientific discussion, the top five most frequently reported terms and phrases among the keywords of the total of these 26 articles of this Research Topic “Microplastics Pollution”, in descending order (in parentheses is the total times of appearance), are as follows: “microplastics” (MPs) (19) > “sediment samples” (11) > “contamination” = “degradation” = “wastewater treatment plants” (5). Based on this overview of keywords, it is noteworthy that the main fields of MP investigation in the relevant literature are that of agricultural soils (agriculture) followed by concerns in wastewater treatment technologies and, at the same time, weak research focus on topics of food and health interest.

## 3. Conclusions

Addressing existing data gaps and prioritizing research in challenging areas are crucial for developing methods and processes that can mitigate the impact of microplastics (MPs) on natural environments. MPs are commonly disposed of and transferred through water channels and trophic transfers, posing risks to human and animal tissues, as well as cultivated soils and natural water sources. Therefore, it is essential to explore ways to either eliminate MPs or render them safe for further consumption and accumulation.

One of the most promising yet challenging areas of future research involves the physical adherence of polymeric-based MPs to organic debris with similar fabrication and texture. This process may stimulate the development of attractive forces and molecular bonds, allowing MPs and organic matter to aggregate under natural outdoor conditions without requiring additional energy or human intervention. By leveraging these natural bonding mechanisms, the agglomerated materials could undergo physical degradation in the environment under two key conditions:

(a) The agglomerating matter should be soluble in aqueous media, including seawater, river water, underground water, runoff, rainfall, or through evapotranspiration cycles.
(b) The agglomerating matter should be dispersed in solution at relatively low concentrations to ensure interaction with natural components and promote degradation in living organisms and environmental systems.

These conditions aim to regulate MP degradation within safe thresholds, such as the lethal concentration 50% (LC50) for living organisms and the carrying capacity of environmental contexts. Ensuring these safe disposal mechanisms would not only address MPs but also other polymeric-fabricated and organic matter that coexist in natural environments.

This future research proposal is conceptualized based on published experimental studies that examine the adsorption of organic-based pesticide molecules onto polymeric absorbents at low concentrations. The adsorption process presents an advantageous, low-energy-consuming mechanism for promoting the adherence of pesticide molecules without excessive energy input. For a more refined fabrication design, researchers should consider the physicochemical properties of hydrophobicity and hydrophilicity, as well as other morphological parameters influencing agglomeration, including pH behavior in aqueous solutions and fluctuations in ionic strength [17,18].

This research approach has the potential to enhance the protection of soil and water sources by removing MPs through interactions with chemically affiliated substances, eliminating the need for human intervention or additional energy consumption. Furthermore, it may facilitate the biomagnification of MPs and associated chemicals from soil and aquifers through trophic transfers [19,20,21]. Rather than focusing solely on MP recovery—which could complicate treatment due to safety concerns in mechanical collection and large-scale environmental storage—this research prioritizes natural compatibility and degradation via bio-modular synergistic processes.

This approach aligns with the study by Jiahui Gu et al. (List of Contributions (24)), which highlights the negative effects of MPs on rainfall infiltration and soil water retention, further supporting the recommendation for their safe disposal in natural environments.
